# Dangers of Chloroform

**Published:** 1848-04

**Authors:** 


*Dangers of Chloroform.*—We have of late seen several notices of accidents attending the use of chloroform. In two instances it appears to have produced fatal consequences. In one of the instances referred to, the patient was operated upon for *fistula in ano* while insensible from the inhalation of chloroform, and died shortly afterward. He had previously been operated upon for the same affection while under the influence of the anæsthetic agent. Dissection revealed pulmonary disease, but this would not account for the sudden death, irrespective of the chloroform. This case occurred in New-York. The other case occurred in Cincinnati. In the latter, the chloroform was administered by a Dentist. On a post-mortem examination conducted by several physicians, among whom was Dr. Lawson, editor of the Lancet, the organs were found free from any lesions which would account for sudden death. These two are the only instances of fatal consequences which have come to us in an authenticated shape. But even a single instance, coupled with the fact that the agent is not infrequently found to produce alarming symptoms, should enforce the propriety of great caution and discrimination in its use. An overweening confidence in its safety has doubtless led to its injudicious employment in many cases. From the tendency of the mind to extremes, it is to be feared, perhaps, that these, and other accidents may lead to its premature abandonment, or to restriction of its use within too narrow limits. A *juste milieu* is desirable, and this is to be established upon the results of rational, careful experience.

